# Detection and characterization of autoreactive memory stem T-cells in children with acute immune thrombocytopenia

**DOI:** 10.1007/s10238-024-01386-0

**Published:** 2024-07-15

**Authors:** Asmaa M. Zahran, Omnia H. El-Badawy, Hayam Mahran, Eman Gad, Khaled Saad, Salma G. Morsy, Ahmed Makboul, Zeinab Albadry M. Zahran, Amira Elhoufey, Hamad Ghaleb Dailah, Khalid I. Elsayh

**Affiliations:** 1 Department of Clinical Pathology, South Egypt Cancer Institute, Assiut University, Assiut, Egypt; 2 Department of Medical Microbiology & Immunology, Faculty of Medicine, Assiut University, Assiut, Egypt; 3 Department of Pediatrics, Faculty of Medicine, Assiut University, Assiut, Egypt; 4 Department of Cancer Biology, South Egypt Cancer Institute, Assiut University, Assiut, Egypt; 5 Department of Clinical Pathology, Faculty of Medicine, Assiut University, Assiut, Egypt; 6 Department of Community Health Nursing, Alddrab University College, Jazan University, 45142 Jazan, Saudi Arabia; 7 Research and Scientific Studies Unit, College of Nursing, Jazan University, Jazan, Saudi Arabia

**Keywords:** Memory T-cells, children, acute immune thrombocytopenia

## Abstract

Primary immune thrombocytopenia (ITP) is an acquired autoimmune disorder characterized by an isolated decrease in platelets below 100 × 10^9^/l after the exclusion of other conditions associated with thrombocytopenia. We investigated the role of different memory T-cell subsets, including T stem cell memory (T_SCM_), in children diagnosed with primary ITP and its association with therapeutic duration. This case–control study included 39 pediatric patients with acute ITP admitted to the Children's Hospital at Assiut University. Using a FACSCanto flow cytometer, CD8 + and CD4 + T-lymphocytes were gated. Five different subsets were characterized in each of these cells according to CD45RO and CD45RA expression. Afterward, gating was performed based on CCR7, CD95, and CD27. Examination of the CD8 + T cells subpopulation showed that Central memory T (T_CM_) and CD8^+^ Naïve T (T_N_) cells were significantly lower in ITP patients than in healthy children (*p* < 0.0001) and (*p* = 0.01), respectively. In addition, CD8 + T_EMRA_ was significantly higher in ITP children than in controls (*p* = 0.001). CD4 + T_CM_ cells were significantly lower in the ITP patient group (*p* = 0.04). However, CD4 + T_EM_ was significantly higher in patients than controls (*p* = 0.04). Our research found that ITP patients had an imbalance in the ratio of CD4^+^ to CD8^+^ T cells in the peripheral blood and that T_CM_ cells may be involved in the pathogenetic mechanism of ITP. T_CMs_ could help in prediction of patients with higher risk of developing ITP.

**Supplementary Information:**

The online version contains supplementary material available at 10.1007/s10238-024-01386-0.

## Introduction

Primary immune thrombocytopenia (ITP) is an acquired autoimmune disorder characterized by an isolated decrease in platelets below 100 × 10^9^/l after the exclusion of other conditions associated with thrombocytopenia. ITP often affects children between two and five years old at a frequency of 4.2 per 100,000 children [1–3]. Based on previous research, aberrant humoral immunity results from the body producing anti-platelet antibodies, causing platelet destruction through the mononuclear-macrophagocyte system [1–3]. Additionally, the loss of platelets is brought on by the damage to and malfunction of bone marrow megakaryocytes. However, only 50 to 70 percent of ITP patients have anti-platelet antibodies [4]. Therefore, the absence of such antibodies does not rule out the diagnosis. It is now proved that cellular immunity contributed significantly to the development of ITP [5]. However, the exact pathogenetic pathways are not fully understood.

After the immune response is triggered by the antigen and resolved, the naive T-cells can produce several subgroups of memory T-lymphocytes with variable genetic expression, phenotype, and anatomical distribution [6]. In addition, the concept of immunological memory is a fundamental characteristic of the adaptive immune system that recalls a particular antigenic exposure and subsequently mounts improved (immediate and effective) responses upon re-exposure [7].

Accordingly, Memory T-lymphocytes are generally classified as central memory (TCM), effector memory (TEM), and CD45RA + effector memory (TERA). These memory T-lymphocyte subsets have short half-lives and a high rate of turnover [8].

Another subset, T stem cell memory (TSCM) cells, possess self-renewal capability and act as a long-lived memory precursor that resembles stem cells [9], where they can differentiate into all memory subsets, including TCM cells [7]. Additionally, they share the same distribution recirculation patterns and keep genes expressed by TN cells [7]. TSCM cells show a CD45RA + CCR7 + CD95 + phenotype and significantly contribute to the development of autoimmune disorders by acting as a source of autoreactive effector T-lymphocytes that support the persistent damage of certain tissues [10].

The balance between CD8 + and CD4 + T lymphocyte frequency is crucial to maintain effective function in the immune system. An abnormal CD4 + / CD8 + ratio has been linked to a variety of illnesses, including malignancies, autoimmune, and infectious disorders [11]. Furthermore, a direct relationship was observed between the frequencies of CD4 + and CD8 + T stem cell memory (TSCM) cells in conditions such as systemic lupus erythematosus (SLE). Additionally, the initial frequency of CD8 + TSCM cells at the time of diagnosis was linked to the effectiveness of immunosuppressive therapy in these cases [11].

Regarding ITP, an increased percentage of CD8 + T cells was associated with increased platelet destruction [12]. We previously reported that The CD4 + /CD8 + ratio was significantly decreased in ITP pediatric patients and that CD8 + cells could be a prognostic marker in these patients [13]. However, the connection between TSCM and ITP in pediatric patients has not yet been documented. Understanding how TSCMs are generated and maintained in ITP patients is essential to advance their therapeutic potential and reduce their adverse effects. In the present study, we investigated the role of different memory T cell subsets, including TSCMs, in children diagnosed with ITP and its association with the therapeutic response.

## Materials and methods

### Study design

This is a case–control study that included 39 children with acute ITP admitted to the Pediatric Clinical Hematology Unit of Children's Hospital in Assiut University. Patients were recruited from January 2021 to the end of November 2021. Twenty healthy children were included in the study as a control group. The control group consisted of children of the same age and sex with good general health status, including the absence of chronic or acute medical conditions and normal platelet count.

The diagnosis was made by evaluating the platelet count (below 100 × 10^9^ cells/L) for less than one year. Newly diagnosed ITP is defined as a disease diagnosed within three months; however, persistent ITP is a disease with a duration between 3 and 12 months. Chronic ITP is defined as thrombocytopenia continuing beyond one year [14] No other hematological abnormalities or organomegaly were seen. The exclusion criteria were secondary thrombocytopenia, including infections, pediatric immunodeficiency disorders, and connective tissue diseases such as SLE, malignancies, drug-induced and congenital thrombocytopenia.

Patients underwent a thorough evaluation that included a detailed medical history, with particular emphasis on prior medications, bleeding symptoms and signs, ITP grading, comprehensive physical examinations, and standard laboratory assessments.

Management was performed according to the ITP grade of severity [15]. Patients were classified based on their response to treatment into two groups: patients with short duration, who recovered before three months, and patients with long duration, who did not recover before three months [16]. Complete remission or complete response to treatment was assessed based on platelet count ≥ 100 × 10^9^/L with no clinically relevant bleeding [17]. Patients received treatment according to the American Society of Hematology 2019 guidelines for immune thrombocytopenia [18].

## Samples collection

Two ml of peripheral venous blood was withdrawn from ITP patients before therapy and controls in ethylenediaminetetraacetic acid (EDTA) vacutainer blood collection tubes. Peripheral blood samples were obtained for routine and flow cytometry studies.

## Flow cytometry

One hundred microliters (µl) of whole blood underwent incubation with specific antibodies under the following conditions: 10 µl of fluoroisothiocyanate (FITC)-conjugated anti-CD27, 10 µl of phycoerythrin (PE)-conjugated anti-CD8, 10 µl of PE-cyanine 7 (PE-CY7)-conjugated anti-CD45RO, 10 µl of allophycocyanin (APC)-conjugated anti-CD45RA, 10 µl of APC-H7-conjugated anti-CD4, 10 µl of PerCP-Cy5.5-conjugated anti-CCR7, and 10 µl of V500-conjugated anti-CD95. This incubation occurred for 15 min at a temperature of 4 ℃ in a light-protected environment. All the monoclonal antibodies used in this process were procured from Becton Dickinson (BD, CA). Subsequently, red blood cell (RBC) lysis buffer was introduced, followed by centrifugation at 2500 revolutions per minute. The resulting pellet was washed with phosphate-buffered saline (PBS). An isotype-matched negative control was utilized to identify any background staining signals present in each sample.

**Fig. 1:**
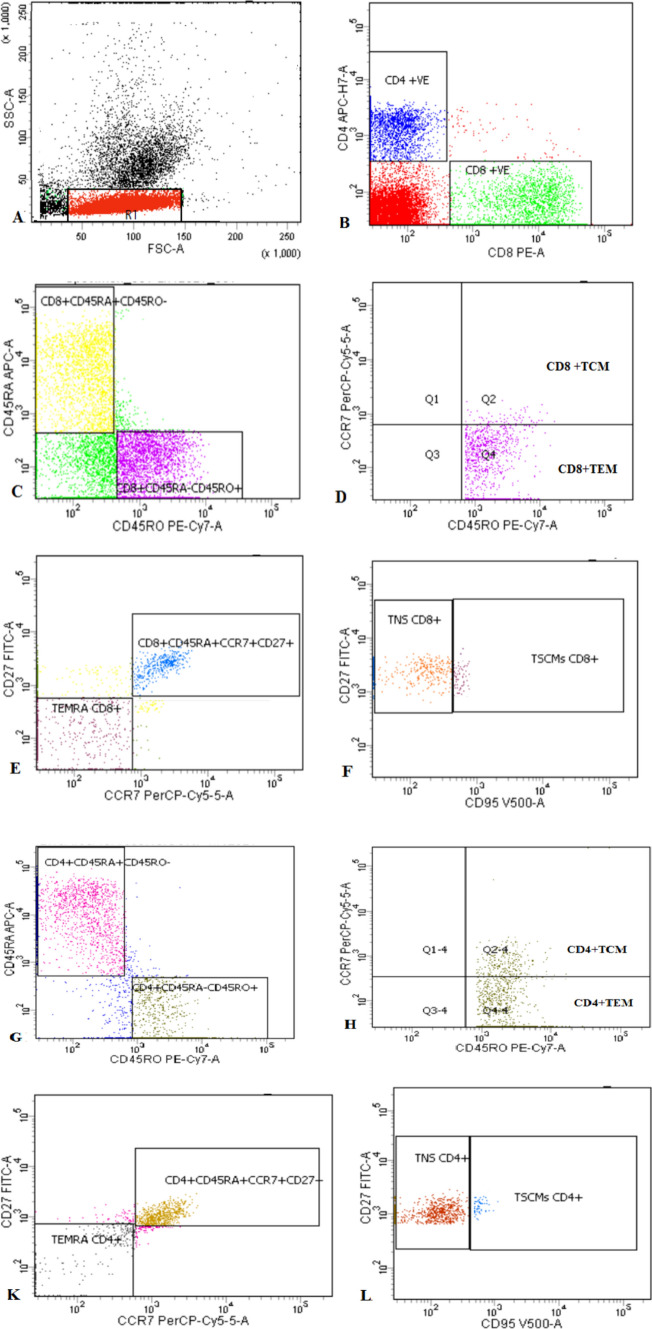
Flow cytometric detection of T lymphocyte subsets. **A:** Lymphocytes were gated based on their characteristics on forward and side scatter histogram. **B:** Then CD4 + cells and CD8^+^ cells were assessed on lymphocytes and then gated for further analysis **C**–**F:** CD8^+^ T cells were subdivided based on characteristic expression patterns of CD45RA, CD45RO, CD27, CCR7 and CD95 into: TCM; CD8^+^ CD45RO^+^CCR7^+^. TEM;CD8^+^ CD45RO^+^ CCR7^−^. TEMRA;CD8^+^ CD45RO^−^ CD45RA^+^ CCR7^−^ CD27^−^. TN;CD8^+^ CD45RO^−^ CD45RA^+^ CCR7^+^ CD27^+^ CD95^−^. TSCM;CD8^+^ CD45RO^−^CD45RA^+^ CCR7^+^ CD27^+^ CD95^+^ **G**–**L**:The same was done for CD4 + T cells

Approximately 100,000 events were recorded for each sample, and data analysis was carried out using the FACSCanto flow cytometer, with analysis facilitated by the FACS DIVA 7.0 software (BD). Lymphocytes were selectively chosen based on their light scatter properties. CD4 + and CD8 + T cells were subsequently gated, and within each of these cell populations, five distinct subsets were delineated based on the expression of CD45RA and CD45RO. Further gating was performed based on CD27, CCR7, and CD95 expression. These identified subsets encompass the following categories: TN (naive T cells, CD45RO − CD45RA + CCR7 + CD27 + CD95 −), TSCM (T memory stem cells, CD45RO − CD45RA + CCR7 + CD27 + CD95 +), TCM (central memory T cells, CD45RO + CD45RA-CCR7 +), TEM (effector memory T cells, CD45RO + CD45RA-CCR7 −), and TEMRA (effector memory T cells re-expressing CD45RA, CD45RO − CD45RA + CCR7 − CD27 −) Fig 1 .

## Statistical analysis

The statistical package for social sciences (SPSS), version 16, was employed for statistical analysis. Descriptive statistics were computed for the variables, including mean values and standard errors of the mean. Due to the small sample size and the potential presence of outliers in some variables, group differences were assessed for statistical significance using the Mann–Whitney analysis. A *p*-value less than 0.05 was deemed statistically significant. Pearson’s correlation analysis was used to investigate the relationships between variables. Simple regression analysis was performed using JMP Pro 16 software (JMP, SAS Institute, North Carolina). Figures were generated using GraphPad Prism (GraphPad Software, San Diego, CA).

## Results

### Basic demographic and laboratory characteristics of the studied groups

The present study was conducted on 39 ITP patients, 18 males and 21 females, and 26 controls. The mean age of ITP patients and controls was 6.6 ± 0.7 and 6.96 + 0.53, respectively. Cases and controls were age and sex-matched. Demographic and laboratory data of patients and controls were shown in Table 1.

**Table 1:** Demographic and laboratory data of the studied groups

Variable	Patients	Patients	*p-*value
Age (years)			
Mean ± SE	6.6 ± 0.7	6.96 + 0.53	0.2
Sex number (%)			
Males	18 (46.2%)	14 (38.4%)	0.7
Females	21 (53.8%)	16 (61.5%)	
Weight (Kg)	21.4 ± 2	23.1 ± 3.5	0.5
Hematological parameters			
Platelets (× 10^6^/L)	49.5 ± 6	267.63 + 12.54	< 0.0001*
MPV (fl)	8.4 ± 0.3	6.08 ± 0.5	< 0.0001*
TLC (× 106/L)	8 ± 0.5	7.3 ± 0.3	0.3
Hemoglobin (g/dL)	11.4 ± 0.2	12.1 ± 0.5	0.2

A *p-*value less than 0.05 is significant

## CD8 and CD4 imbalance in ITP patients

Our results showed that the mean percentage of CD8^+^ T cells in ITP children was significantly higher than in healthy control (28 ± 1 vs. 24.2 ± 0.9; *p* = 0.01). However, the mean percentage of CD4 + T cells was signifying lower in patients than in controls (29.8 ± 1 vs. 37.6 ± 5, *P* < 0.0001). In addition, CD4/CD8 ratios were significantly lower in ITP children when compared to controls (1 ± 0.06 vs.1.6 ± 0.07, *p* < 0.0001).

## CD8 + population in ITP

Analysis of the CD8^+^ T cells subpopulation showed that the mean percentages of CD8^+^ Naïve T (T_N_) and Central memory T (T_CM_) were significantly lower in ITP patients than in controls (20 ± 2 vs. 31.4 ± 2, *p* < 0.0001) and (13 ± 0.5 vs. 16.8 ± 1, *p* = 0.01), respectively. In addition, the mean percentage of highly differentiated CD8 + T_EMRA_ was significantly higher in ITP children than in healthy control (19.9 ± 1 vs. 14 ± 1, *p* = 0.001). However, there was no significant difference between patients and controls in the mean percentages of CD8^+^CD45RA^+^, CD8^+^CD45RO^+^, CD8^+^ T_SCM_, and CD8^+^ T_EM_ T-cell subpopulations. (Table 2) (Figure S1 in supplementary file).

**Table 2:** Characterization of CD8 + T cells subpopulation in ITP patients and controls

Cells (%)	Patients	Controls	*p-*value
CD8^+^ T cells	28 ± 1	24.2 ± 0.9	0.01*
CD8^+^CD45RA^+^ T cells	43.5 ± 1	45.9 ± 2	0.4
CD8^+^CD45RO^+^ T cells	45 ± 1	43.7 ± 2	0.4
CD8^+^ T_N_	20 ± 2	31.4 ± 2	< 0.0001*
CD8^+^ T_SCM_	3.8 ± 0.3	3.2 ± 0.2	0.6
CD8^+^ T_CM_	13 ± 0.5	16.8 ± 1	0.01*
CD8^+^ T_EM_	30 ± 1	26.9 ± 1	0.1
CD8^+^ T_EMRA_	19.9 ± 1	14 ± 1	0.001*

(TN), Naïve T; (TSCM), stem cell memory T; (TCM), central memory T; (TEM), effector memory; (effector memory re-expressing CD45RA), highly differentiated TEMRA

A *p-*value less than 0.05 is significant

## CD4 + population in ITP

Our results showed that the mean percentage of CD4^+^CD45RA^+^, CD4^+^CD45RO^+^, CD4^+^ T_N_, CD4^+^ T_EMRA_, and CD4 + T_SCM_ T-cell subpopulations was comparable between patients and controls (Table 3). CD4 + Central memory T (T_CM_) cells were significantly lower in the ITP patient group (25 ± 1 vs. 29.8 ± 2, *p* = 0.04). However, CD4^+^ T_EM_ was significantly higher in patients than in healthy controls (14.7 ± 1 vs. 11.6 ± 0.8, *p* = 0.04) (Figure S2 in supplementary file).

**Table 3:** Characterization of CD4 + T cells subpopulation in ITP patients and controls

Cells (%)	Patients	Controls	*p-*value
CD4^+^ T cells	29.8 ± 1	37.6 ± 5	< 0.0001*
CD4^+^CD45RA^+^ T cells	45.9 ± 1	47.4 ± 2	0.4
CD4^+^CD45RO^+^ T cells	41.7 ± 1	40.8 ± 1	0.7
CD4^+^ T_N_	37.5 ± 1	37.6 ± 1	0.9
CD4^+^ T_SCM_	2.7 ± 0.3	2 ± 0.2	0.4
CD4^+^ T_CM_	25 ± 1	29.8 ± 2	0.04*
CD4^+^ T_EM_	14.7 ± 1	11.6 ± 0.8	0.04*
CD4^+^ T_EMRA_	3.7 ± 0.4	3 ± 0.3	0.5

(TN), Naïve T; (TSCM), stem cell memory T; (TCM), central memory T; (TEM), effector memory; (effector memory re-expressing CD45RA), highly differentiated TEMRA)

A *p-*value less than 0.05 is significant

## Changes in T cell subsets regarding ITP duration

Comparing T cell subsets among patients with ITP duration less than and more than three months revealed a significant increase in CD8^+^ T cells and CD8^+^ T_SCM_ subsets in patients with shorter duration. In addition, patients with longer ITP duration had a significant increase in CD4^+^ T_CM_ cells. There was no significant difference regarding other T cell subsets (Table 4) (Figure S3 in supplementary file).

**Table 4:** Percentages of T cell subsets according to the duration of thrombocytopenia

Cells (%)	< 3 months	> 3 months	*p-*value
CD8^+^ T cells	30.8 ± 1	25.7 ± 1	0.01
CD8^+^CD45RA^+^ T cells	41 ± 1	45.2 ± 2	0.1
CD8^+^CD45RO^+^ T cells	47.2 ± 2	43.9 ± 1	0.1
CD8^+^ T_N_	18.6 ± 2	20.8 ± 2	0.8
CD8^+^ T_SCM_	4.8 ± 0.6	3 ± 0.4	0.01
CD8^+^ T_CM_	13.8 ± 0.9	12.5 ± 0.6	0.5
CD8^+^ T_EM_	31.6 ± 2	29.3 ± 2	0.3
CD8^+^ T_EMRA_	18.4 ± 1	21.2 ± 2	0.4
CD4^+^ T cells	31.7 ± 1	28.3 ± 2	0.08
CD4^+^CD45RA^+^ T cells	47.6 ± 1	44.4 ± 2	0.1
CD4^+^CD45RO^+^ T cells	40.2 ± 2	43.3 ± 2	0.2
CD4^+^ T_N_	39.6 ± 2	35.7 ± 2	0.1
CD4^+^ T_SCM_	3.2 ± 0.6	2.4 ± 0.3	0.6
CD4^+^ T_CM_	22.6 ± 1	27.4 ± 2	0.04
CD4^+^ T_EM_	15.3 ± 2	14.2 ± 2	0.5
CD4^+^ T_EMRA_	3.9 ± 0.6	3.7 ± 0.5	0.7
CD4/CD8 ratio	1 ± 0.08	1.2 ± 0.1	0.8

## Correlation of T cell subsets with laboratory parameters and ITP duration

Correlation analysis showed a significant negative correlation between CD8, CD8 TSCMs, and CD4 TNs with platelet count. Moreover, the percentage of CD8 TSCMs was inversely correlated to ITP duration. (Table 5).

**Table 5:** Significant correlations

CD8	• Weight *r* = − 0.3, *p* = 0.046, • Platelets *r* = − 0.5, *p* = 0.001
CD8 T_N_	• Lymphocyte count *r* = − 0.4, *p* = 0.004
CD8 T_SCM_	• Platelets *r* = − 0.4, *p* = 0.008, • Lymphocyte count *r* = 0.3, *p* = 0.04, • Thrombocytopenia duration *r* = − 0.4, *p* = 0.01
CD8 T_EM_	• Monocyte count r = − 0.3, *p* = 0.03
CD4 T_N_	• Neutrophil *r* = − 0.3, *p* = 0.02, • Lymphocyte % *r* = 0.3, *p* = 0.02, • Platelets *r* = − 0.4, *p* = 0.04
CD4 T_SCM_	• Lymphocyte count *r* = 0.3, *p* = 0.03, • Monocyte count *r* = 0.5, *p* < 0.0001
CD4 CD45RO	• Weight *r* = 0.3, *p* = 0.04

In addition, simple regression analysis showed that CD8^+^ and CD8^+^TSCM cells significantly affect the Patient's platelet count (*p* = 0.002 and *p* < 0.0001), respectively (Figure S4 in supplementary file).

## Discussion

The present study thoroughly investigated T cells and their subpopulation in the periphery of the ITP pediatric patient cohort and analyzed the connection between these immunological indicators and therapeutic outcomes [18]. T cells are still in the central stages of anti-platelet autoimmunity, playing a crucial role in its genesis, transmission, and maintenance [19].

Our results demonstrated that ITP patients had an imbalance in T lymphocyte subsets, including a significant decrease in CD4^+^ Th cells and CD4/CD8 ratio with excessive CD8^+^ Tc cells in patients than controls (Fig. 1). Previous studies revealed that ITP’s pathogenesis involves aberrant immunocyte subsets [13, 20, and 21]. Moreover, studies showed that CD8^+^ Tc cell-mediated autoimmunity can occur independently of autoantibody-mediated autoimmunity, where they directly attack platelets and megakaryocytes [22].

A significant increase in CD8^+^ Tc could be due to a prior viral infection, in which the production of platelets displaying viral antigens mounts CD8^+^ Tc cells' cytotoxic response, causing immune thrombocytopenia [23]. Also, T-helper 1 (TH1) bias was demonstrated in ITP, which in turn stimulates CD8^+^ Tc function and IgG production [24]. Aslam et al. [19] and Johnsen [25] found that the total CD4 + /CD8 + ratio is reduced in ITP and improves with illness remission, supporting our results. In addition, A significantly higher percentage of CD8^+^ Tc cells was detected in patients with thrombocytopenia for a brief duration (< 3 months) than in those with prolonged thrombocytopenia > 3 months. This is consistent with the preceding outcomes and may be related to previous viral infections [20, 26]. CD45 isoforms have long been used to distinguish between naive (CD45RA^+^CD45RO) and memory (CD45RACD45RO^+)^ T cells [27]. Other surface markers, such as CCR7 [28], CD27 [29], and CD95 [30], when used along with CD45RA, provide a far more in-depth view of the T cell maturation process [2].

Memory T cells are classified as CD45RACCR7^+^ central memory T (T_CM_) cells that migrate to lymphoid tissues and CD45RACCR7 effector memory T (T_EM_) cells that travel to various peripheral tissue sites. T_CM_ can efficiently differentiate into effector cells during proliferation. Both T_CM_ and T_EM_ subsets release effector cytokines in response to viruses, antigens, and other stimuli, whereas T_CM_ cells have a higher proliferation capability [31]. Several identified populations, such as CD27CD45RA^+^CD197^+^CD95^+^ (now known as T_EMRA_) and CD27^+^CD45RA^+^ CD95^+^ (now known as T_SCM_), were first discovered in CD8^+^ T cells and afterward in CD4^+^ T lymphocytes. Both have Variable effector capacities and considerable changes in response based on the stimulating antigen [32]. In the current study, the percentage of naive T lymphocytes in ITP patients was significantly lower than in healthy controls regarding CD8^+^ cells, given the previously documented lower expression of CD45RA in autoimmune disorders [11]. However, no significant difference was noticed between patients with brief or long ITP duration.

Furthermore, a significant decrease in the percentage of CD4^+^ and CD8^+^ T_CM_ cells with a corresponding high percentage of CD4^+^ and CD8^+^ T_EM_ cells was observed among ITP patients compared to the control group. We suggest that T_CM_ homing may be affected differently by changes in T cell subtypes in ITP patients, together with reduced T cell response. Elevated T_EM_ cells with potent effector capacity have been identified as a potential indicator of abnormal immunity in aplastic anemia [33]. In addition, previous studies showed that exposure to chronic autoantigen seems to favor the development of CD4 + T_EM_ while hindering CD4 + T_CM_ cell formation, such as in chronic infection and different autoimmune diseases [34, 35]. Parallel results were specified by Xu et al. [36], who reported a decrease in T_CM_ cells and excessive aggregation of T_EM_ and T_EF_ cells in patients with acute myeloid leukemia.

Nonetheless, in patients with longer thrombocytopenic duration, we found a significant increase in the percentage of CD4^+^ T_CM._ Moreover, the percentage of CD8^+^ T_EMRA_ was significantly higher in ITP patients than control. T_EMRA_ cells were initially characterized in the context of viral infections and vaccination responses [37, 38]. These cells have expanded cytotoxic characteristics supporting the hypothesis of platelet lysis by CD8^+^ T cells [39]. A prior investigation demonstrated that these cells have the ability to attach to platelets, leading to TCR-mediated activation and subsequent death of the platelets. This mechanism operates independently of antibodies and offers an alternative means of causing platelet destruction. Besides, it was revealed that these cells exhibit multiple functions, including the production of IFN-gamma, TNF-alpha, and granzyme B. Notably, there were no indications of physiological depletion, and these findings were found to be correlated with the activity of immune thrombocytopenia (ITP) disease [40].

Stem cell memory T cells (T_SCM_ cells) were shown to possess the ability to self-renew and exhibit multipotency. This characteristic suggests that TSCM cells could serve as a potential long-term reservoir for T-cell memory throughout an individual's life [41]. At this firm, there was no significant difference in the percentages of CD8^+^ and CD4^+^ T_SCM_ between patients and controls. However, CD8 T_SCM_ was significantly higher in patients with shorter ITP duration and inversely correlated with the platelet count. Since T_SCM_ cells may produce all memory and effector T cell subsets, it was thought that their rising prevalence might contribute to the emergence of autoimmune diseases [11]. In aplastic anemia, higher CD8^+^ T_SCM_ frequency at the time of diagnosis was discovered to be related to greater response. However, increased CD8 + TSCM cells following immunosuppressive therapy was linked to treatment failure and relapse [11], suggesting CD8 + T_SCMs_ might be a potential biomarker.

## Conclusion

Our research found that ITP patients had an imbalance in the ratio of CD4^+^ to CD8^+^ T cells in the peripheral blood and that T_CM_ cells may be involved in the pathogenetic mechanism of ITP. T_CMs_ could help in prediction of patients with higher risk of developing ITP.

## Supplementary Information

Below is the link to the electronic supplementary material.

Supplementary file1 (PDF 366 KB)

## Data Availability

The datasets used and/or analyzed during the current study are available from the corresponding author upon reasonable request.
