# Supplementary figures and images for: Detection and characterization of autoreactive memory stem T-cells in children with acute immune thrombocytopenia
Source: Clin Exp Med. 2024 Jul 15;24(1):158. doi: 10.1007/s10238-024-01386-0 (PMC11247050; doi:10.1007/s10238-024-01386-0)

Figure S 1

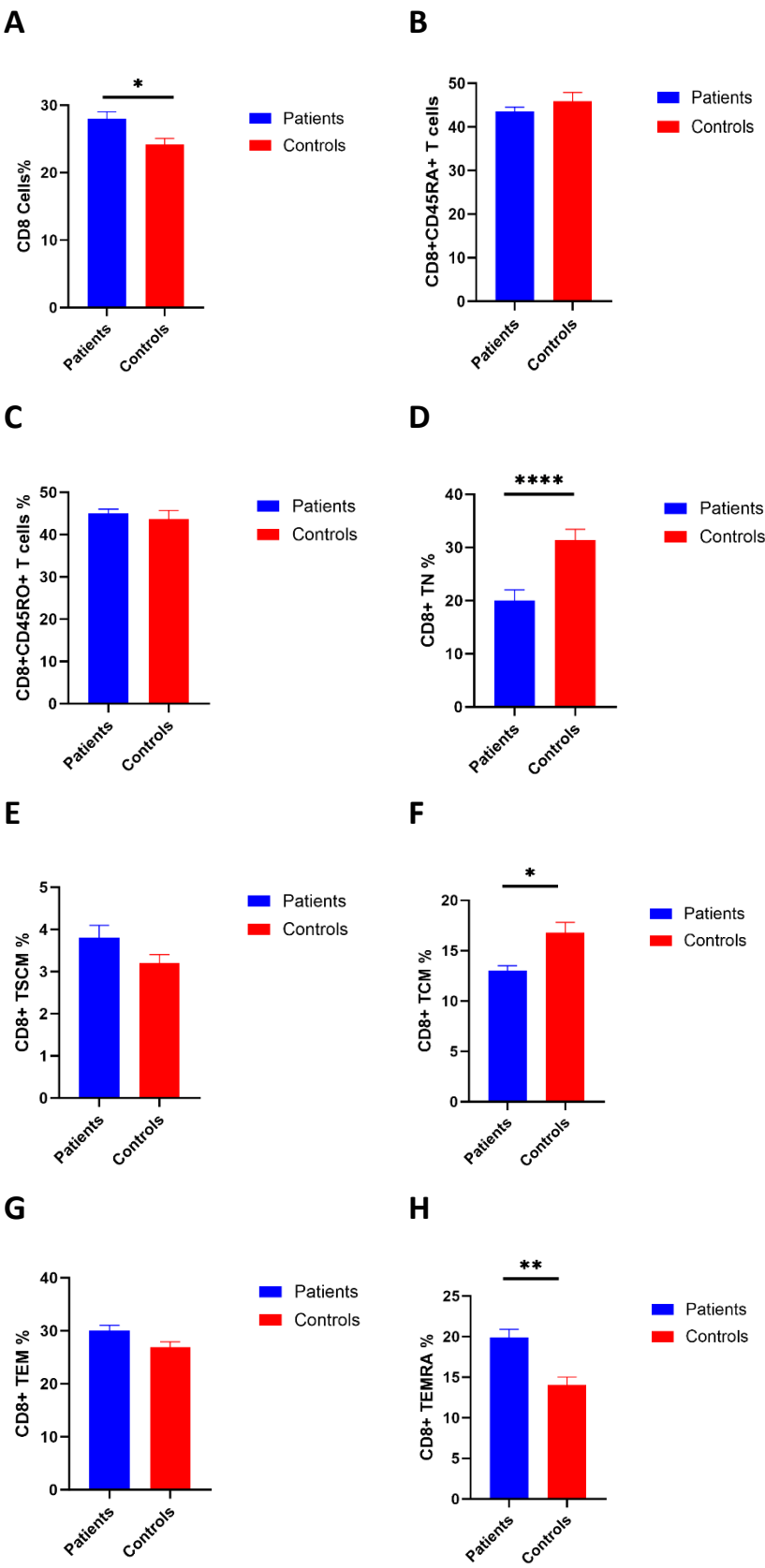

Figure S2

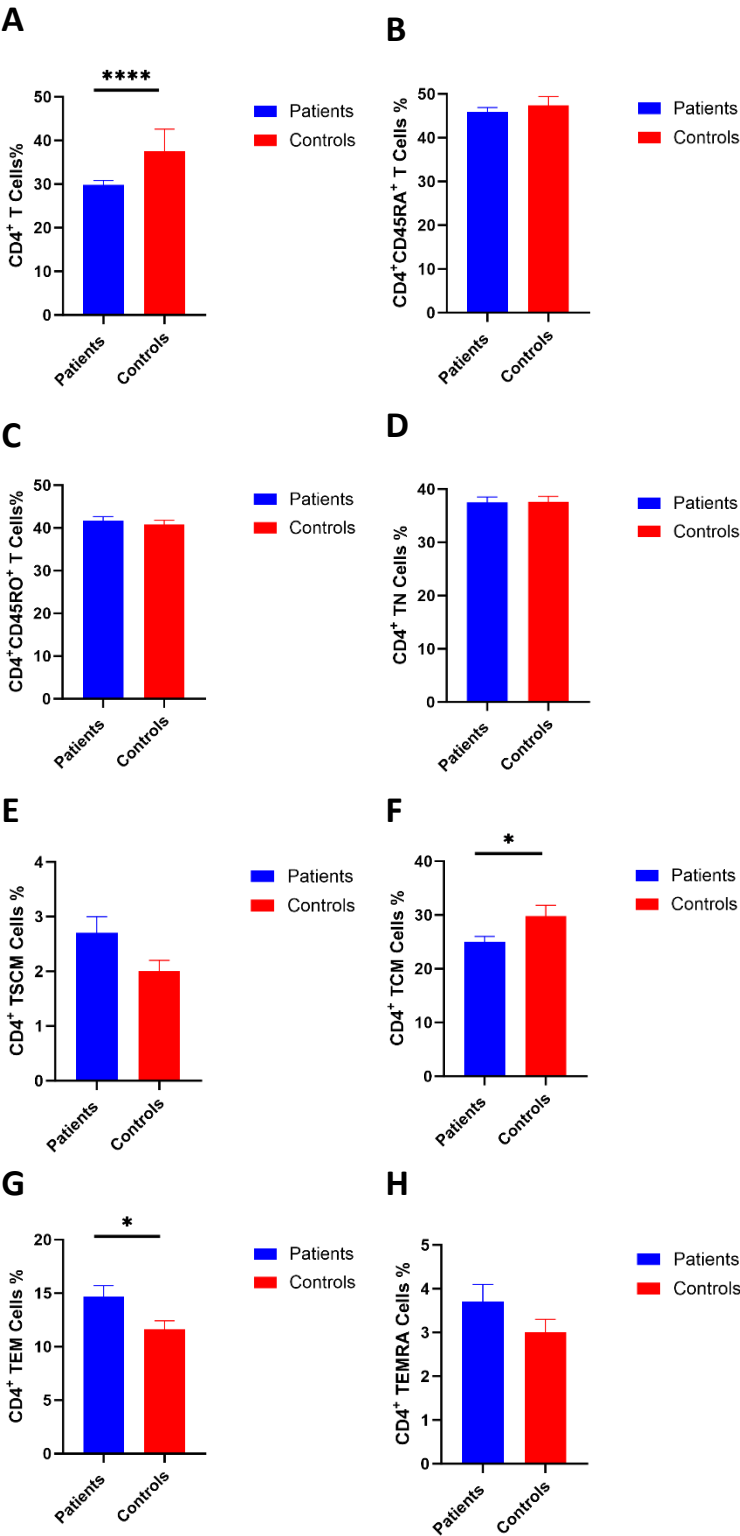

**Figure S3**

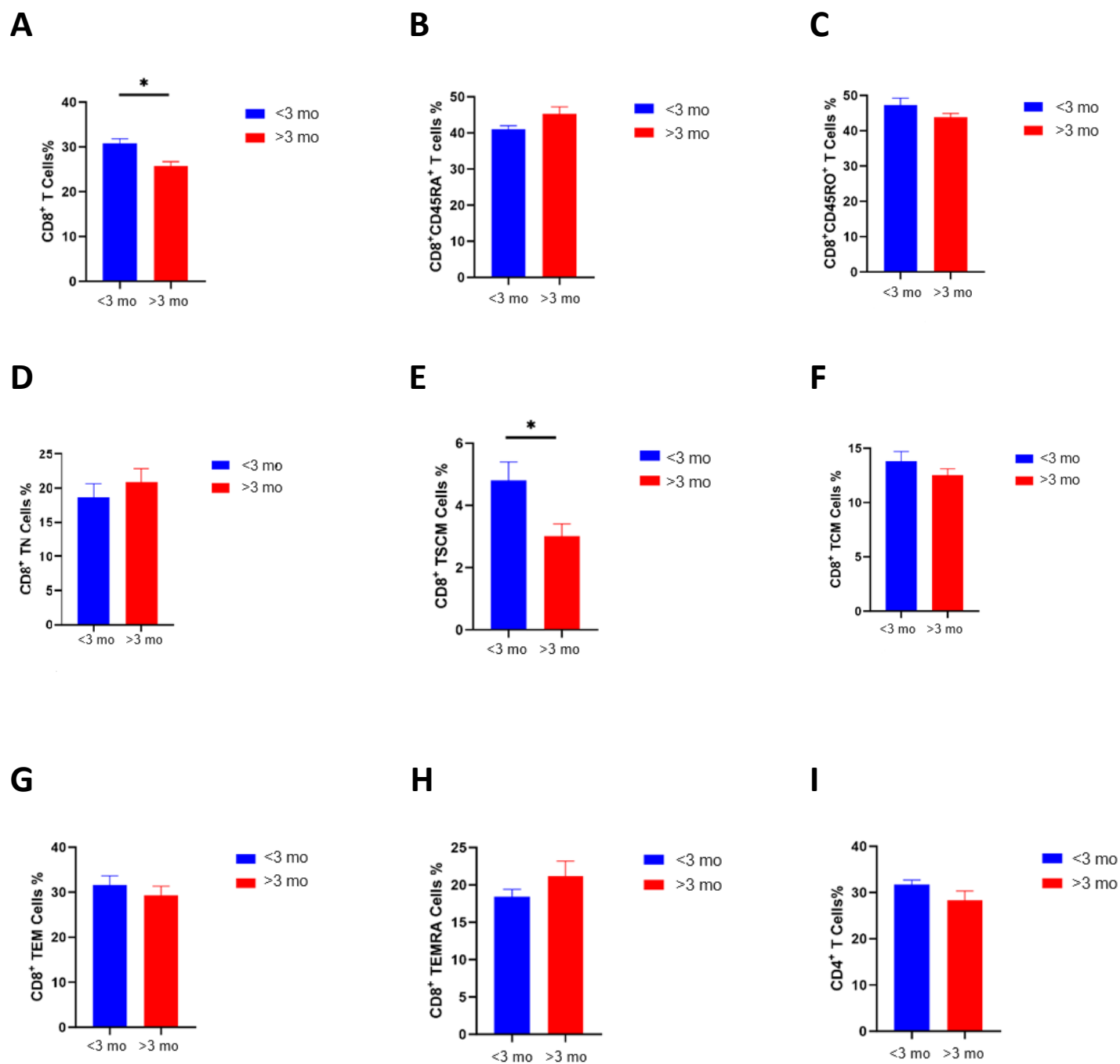

**J**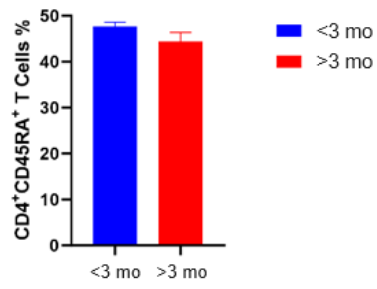**K**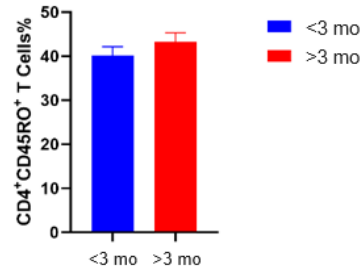**L**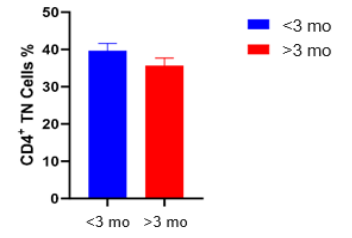**M**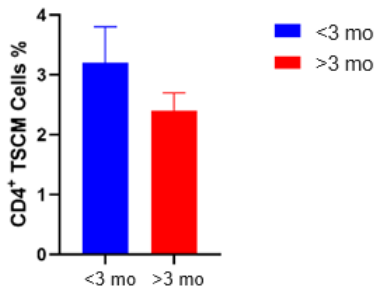**N**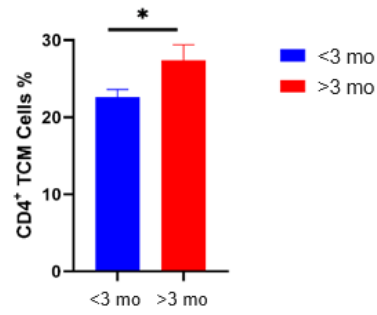**O**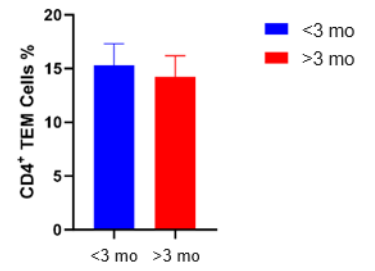**P**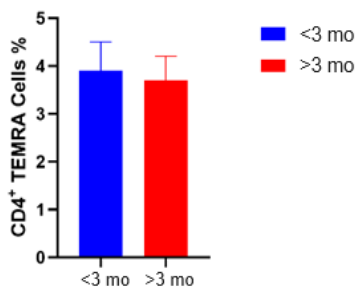**Q**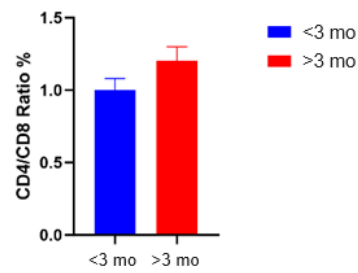

Figure S4

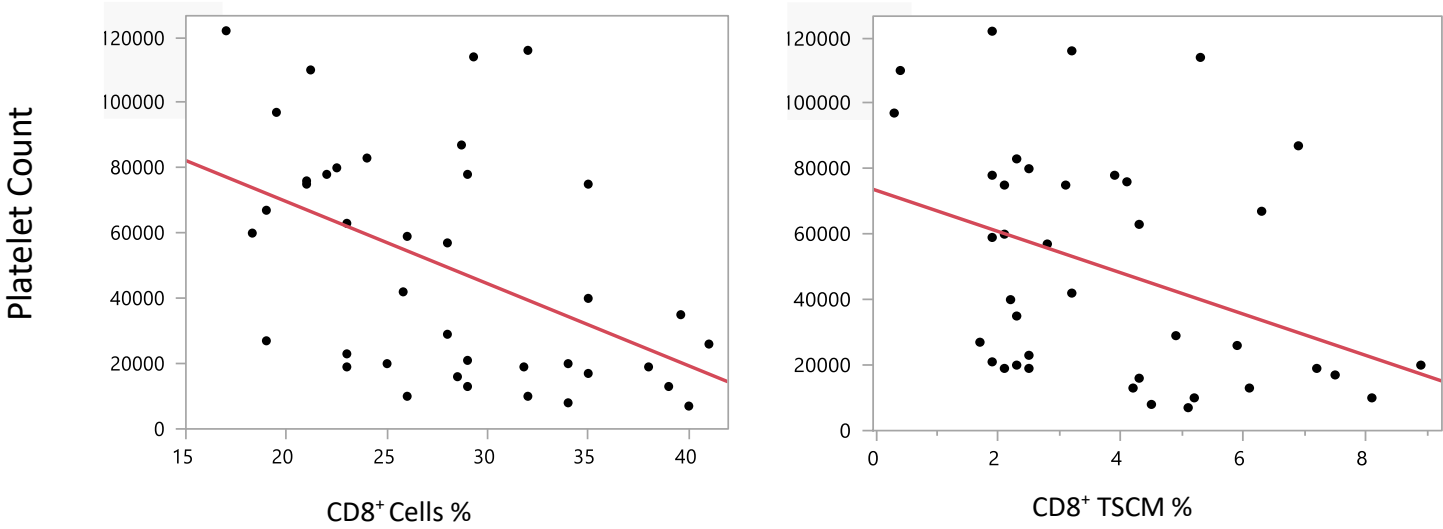

Supplement: Supplementary file 1 — Supplementary file1 (PDF 366 KB) [file 10238_2024_1386_MOESM1_ESM.pdf]
